# The Role of Neutrophil Extracellular Traps in Central Nervous System Diseases and Prospects for Clinical Application

**DOI:** 10.1155/2021/9931742

**Published:** 2021-07-13

**Authors:** Yinghan Guo, Hanhai Zeng, Chen Gao

**Affiliations:** Department of Neurosurgery, Second Affiliated Hospital, School of Medicine, Zhejiang University, Hangzhou, Zhejiang, China

## Abstract

Neutrophil extracellular traps (NETs) are complexes of decondensed DNA fibers and antimicrobial peptides that are released by neutrophils and play important roles in many noninfectious diseases, such as cystic fibrosis, systemic lupus erythematosus, diabetes, and cancer. Recently, the formation of NETs has been detected in many central nervous system diseases and is thought to play different roles in the occurrence and development of these diseases. Researchers have detected NETs in acute ischemic stroke thrombi, and these NETs are thought to promote coagulation and thrombosis. NETs in ischemic brain parenchyma were identified as the cause of secondary nerve damage. High levels of NETs were also detected in grade IV glioma tissues, where NETs were involved in the proliferation and invasion of glioma cells by activating a signaling pathway. Extracellular web-like structures have also recently been observed in mice with traumatic brain injury (TBI), and it was hypothesized that NETs contribute to the development of edema after TBI. This article reviews the effect of NETs on multiple diseases that affect the CNS and explores their clinical application prospects.

## 1. Background

Neutrophils are critical components of the innate immune system and play important roles in central nervous system (CNS) diseases. It is well known that few neutrophils are present in the CNS under physiological conditions due to the brain-blood barrier (BBB). When the CNS suffers from bacterial infection, numerous neutrophils infiltrate the brain parenchyma and cerebrospinal fluid to resist the invasion of bacteria [1]. In addition, neutrophils play crucial roles in some aseptic inflammatory lesions of the CNS. Neutrophils disrupt the blood-brain barrier by releasing matrix metalloproteinases (MMPs), ROS, and elastase [2–7] and contribute to the pathogenic mechanism of diseases such as ischemic stroke (IS), cerebral hemorrhage, and trauma [8–11]. Recently, researchers observed that accumulated neutrophils may also discharge web-like chromatin structures modified with antimicrobial peptides named neutrophil extracellular traps (NETs), which destroy the BBB, lead to the subsequent damage of neurons, and are involved in many CNS diseases [12, 13].

**Figure 1:**
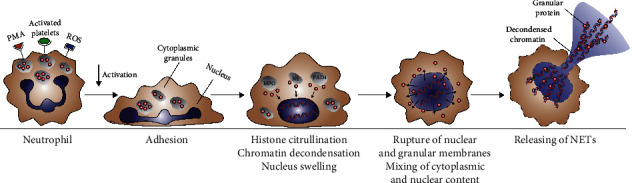
Release process of NETs.

Early in 2004, active neutrophils were found to release web-like structure consisting of decondensed (unwound) DNA decorated with histones and granular proteins, which were termed as NETs [14]. NETs were first discovered in the process of defending against invading microorganisms. By binding microorganisms with web-like structures, NETs immobilize microbes, stop their spread, and provide a high concentration of antimicrobial peptides to kill pathogens such as bacteria [14], fungi [15], and parasites [16]. Crucial steps in the formation of NETs were described in early in vitro studies [17, 18]. Neutrophil nuclei lose their characteristic lobular shape and then swell after stimulation. Data from many researchers show that the citrullination of histones by peptidyl arginine deiminase 4 (PAD4) mediates chromatin decondensation, leading to nuclear swelling [19–22]. It was also suggested that neutrophil elastase (NE) synergized with myeloperoxidase (MPO) to cleave histones and facilitate chromatin decondensation [23]. Subsequently, nuclear and granular membranes decompose, and the decondensed nuclear and granular proteins are mixed in the cytoplasm. Ultimately, the cell membrane breaks, and the mixture of DNA and proteins released into the extracellular compartment (Figure 1). Fuchs et al. showed that this neutrophil death process, named NETosis, depends on the production of ROS by NADPH oxidase (Nox) [18]. In vitro experiments showed that neutrophils stimulated by hydrogen peroxide could induce the formation of NETs. Moreover, either inhibition of NADPH oxidase by diphenylene iodonium or destruction of hydrogen peroxide by catalase shows inhibition of NETs production.

NETosis takes quite a long time and is followed by neutrophil lysis. Interestingly, Pilsczek et al. [24] showed that neutrophils could rapidly expel NETs within minutes without undergoing cell death in response to Staphylococcus aureus. NETs produced by this new mechanism were termed as “vital” NETs. Neutrophils extrude their decondensed chromatin without the release of cytoplasmic contents or cell membrane disintegration. Denucleated neutrophils are still capable of creeping, chasing, and engulfing microorganisms trapped in their web-like structures. Unlike that described by Fuch et al., such NETs are produced in a manner that is independent of Nox. Further studies have shown that calcium influx and mitochondrial ROS release are necessary for the progress of the Nox-independent NET formation. Current studies have shown that the Nox-dependent pathway and Nox-independent pathway are two different ways of NET formation. The activators and subsequent activated kinases required by these two ways are different. Although studies of NET formation have been ongoing for 16 years, there is still no uniform and standardized definition of the mechanism by which NETs are induced.

Although the formation of NETs is one of the main mechanisms of the bactericidal effect of neutrophils, increasing studies have shown that the NETs can also cause adverse effects on the body. On the one hand, antimicrobial peptides such as neutrophil elastase (NE) in NETs cannot only kill pathogens but also cause tissue damage. On the other hand, histone and other substances of NETs can act as autoantigens to cause autoimmune reaction [14]. It has been reported that excessive NET formation may contribute to the pathogenesis of systemic lupus erythematosus [25–27], atherosclerosis [28], thrombosis [29–31], lung injury [32], diabetes [33], and tumors [34, 35]. As described later in this review, NETs are also implicated in CNS diseases, including stroke, Alzheimer's disease, and multiple sclerosis (MS).

## 2. NETs in Stroke

IS is the primary cause of adult disability worldwide. Local thrombosis or peripheral circulatory clots migrate into the brain and block the blood supply of the brain, leading to the formation of an ischemic core and surrounding salvageable ischemic penumbra. In 2017, Laridan et al. [36] demonstrated the presence of NETs in ischemic stroke thrombi for the first time. Inspection of thrombi extracted from cerebral circulation in patients with IS showed the presence of citrullinated histones, which are hallmark of NET formation [36, 37], indicating that NETs may contribute to the pathogenesis of IS. Although the role of NETs in thrombosis has long been described [30, 31], it is still unclear whether and how NETs promote coagulation and thrombosis in IS. Experiments by Peña-Martínez et al. [38] indicated that activation of neutrophils through platelet toll-like receptor 4 (TLR4) could result in NETosis and therefore the formation of arterial thrombi in the brain. Zhou et al. documented neutrophil activation and NET release at the site of plaque rupture in internal carotid artery (ICA) occlusion patients [39]. NETs, which bind platelet-derived microparticles (PMPs) and clotting factors, could act as assembly platforms for atherothrombosis by promoting the formation of thrombin and fibrin. Furthermore, NET-associated proteases and citrullinated histones may damage endothelial cells and augment their coagulant activity through the induction of exposed phosphatidylserine (PS) and tissue factor (TF) expression in endothelial cells [39].

Researchers found that the content of web-like structures in thrombi may impair t-PA-induced thrombolysis in IS. The potential mechanism may be as follows: by forming a ternary complex of DNA, fibrinolytic protein, and fibrin, the extracellular decondensed chromatin in NETs wraps fibrin tightly and impedes fibrinolytic protein-mediated fibrin clot disintegration, resulting in fibrinolysis impairment [40]. Additionally, histone-DNA complexes can improve the stability and rigidity of fibrin in thrombi, leading to prolonged fibrinolysis time [41]. Peña-Martínez et al. [38] simulated t-PA resistance by inducing platelet-rich fibrin-free thrombi in mice. The application of DNase-I, which promotes NETs degradation, recanalized the occluded vessels, confirming that NETs could exert detrimental effects on IS thrombolysis. It was recently reported that recombinant DNase 1 accelerated t-PA-induced thrombolysis in vitro [36], whereas DNase 1 alone was ineffective [37], suggesting that both fibrin and neutrophil-derived extracellular DNA must be targeted to induce successful thrombolysis.

It has been widely recognized that following primary ischemic injury, neuroinflammation promotes secondary exacerbation in the ischemic brain. As one of the first immune cells to infiltrate brain lesions [42], neutrophils play a prominent role in aseptic inflammation. Kim et al. [43] observed spatiotemporal progression of NETosis in the mouse brain after middle cerebral artery occlusion (MCAO). NETosis was detected in peripheral blood and meningeal vessels after 12 hours of MCAO, and neutrophils infiltrated the brain parenchyma and released NETs 1 day after MCAO. A study displayed NETosis inside and around blood vessels [44], combined with the established evidence of BBB destruction caused by neutrophil elastase [45], indicating that neutrophils could attack the BBB by releasing decondensed chromatin lined with proteases. There was evidence that digesting NETs with DNase 1 significantly reduced BBB damage [46].

Early studies have suggested that the main cause of delayed neuronal damage after cerebral ischemia is the migration of neutrophils to the brain parenchyma and the release of a large number of proteases. Recently, researchers found citrullinated histone H3 (CitH3) and extracellular DNA fibers in the ischemic brain, suggesting that NETs play a role in neurological dysfunction after ischemia [44, 46]. Allen et al. identified a novel neuroinflammatory mechanism in the ischemic brain: by migrating across the BBB, neutrophils cause neuronal death by altering their phenotypes and releasing decondensed chromatin and proteases [12]. Notably, interleukin-1 (IL-1) plays a key role in cerebrovascular activation, neutrophil recruitment, and transendothelial migration [12]. Experiments by Kim and colleague [43] suggest that high-mobility group box-1 (HMGB1), a prototypic danger-associated molecular pattern (DAMP), is involved in NET-mediated neuronal damage in the ischemic brain. On the one hand, HMGB1 is released from neuronal nuclei after acute damage and serves as a mediator that leads to the formation of NETs in neutrophils. On the other hand, after neutrophil activation, HMGB1 in neutrophil nuclei is extruded to the extracellular space during NETosis and therefore exacerbates neuroinflammation by further recruiting and activating neutrophils and other immune cells.

During the convalescence of cerebral ischemia, neovascularization and vascular remodeling are critical for the repair of the brain function. Kang et al. [46] discovered that PAD4 was markedly increased in the ischemic peripheral cortex, leading to a reduction in neovascularization by the increased release of NETs. Consistent with these observations, vascular branches, microvascular length, and perfused capillary length were increased in the ischemic brains of mice treated with DNase 1.

**Figure 2:**
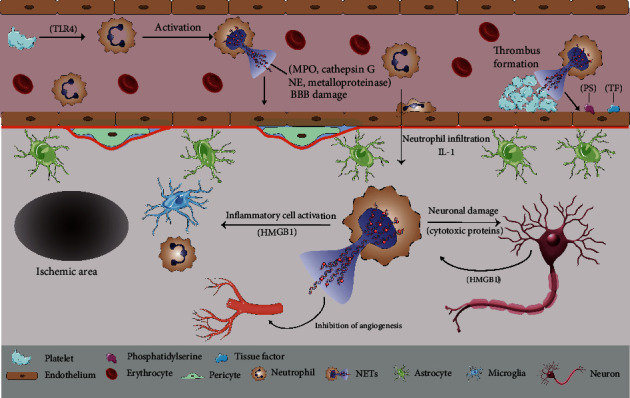
Contribution of NETs to IS. After forming in blood vessels, NETs can cause blood-brain barrier damage, which may be related to the granular protein it contains. Intravascular NETs can enhance coagulation activity and act as assembly platform to promote thrombosis. After transferring to brain parenchyma by changing phenotype, neutrophils release NETs, which can directly damage neurons through cytotoxic proteins or aggravate neuroinflammation by activating inflammatory cells.

Figure 2 Contribution of neutrophil extracellular traps to ischemic stroke.

As another devastating form of stroke, intracerebral hemorrhage (ICH) is a severe acute cerebrovascular event with high mortality and poor prognosis. Despite the increased intracranial pressure (ICP) and brain tissue damage caused by the accumulation of blood within the brain parenchyma, ICH is also characterized by inflammation-mediated brain damage [47, 48]. Consistent with IS, neutrophils are among the first inflammatory cells to infiltrate the brain tissue in ICH. An abundance of neutrophil accumulation in the ICH core and hematoma border has been documented in experimental intracerebral hemorrhage [47]. By releasing matrix metalloprotease 9 (MMP-9), neutrophils can cause BBB destruction and axonal damage after ICH [48]. However, there is a lack of data on NETosis in cerebral hemorrhage. Recently, an animal experiment first confirmed the presence of NETs in ICH by the colocalization of DAPI, histone H3, and MPO [49] in the ICH rat brain. An in vitro experiment showed that hemin, a heme-related molecule produced by the hemolysis of red blood cells, can activate neutrophils and induce morphological changes, degranulation, and NET release in neutrophils, which may explain how NETs are induced in ICH [50]. However, it is still unclear whether and how NETs cause damage to the blood-brain barrier and exert detrimental effects on neurons in ICH. Further experiments by Tan et al. demonstrated that DNase 1 could promote t-PA-induced hematoma fibrinolysis, thereby relieving brain swelling, reducing neuronal death, and improving functional prognosis in rats with ICH [49].

## 3. NETs in Alzheimer's Disease (AD)

AD is a chronic progressive neurodegenerative disorder characterized by memory deterioration and cognitive impairment. The pathological characteristics of AD include senile plaques with amyloid beta (A*β*) peptide deposition, neurofibrillary tangles containing hyperphosphorylated neuronal tau protein, and synaptic dysfunction [51–53]. Additionally, neuroinflammation is thought to play an important role in the pathological process of AD because leukocytes such as lymphocytes, monocytes, and neutrophils have been discovered in the brains of these patients [54–56]. Studies have shown that neutrophil depletion can improve the cognitive function and reduce AD-associated pathological damage in AD model mice [56, 57]. NETs-producing neutrophils discovered in the parenchyma and blood vessels of AD model mice may support the idea that NETs, which are involved in neutrophil-mediated chronic neuroinflammation, and promote the pathogenesis or development of AD.

On the one hand, destruction of the BBB in AD model mice has been widely reported [58, 59]. Current evidence shows that BBB permeability precedes senile plaque formation and cognitive deficits, suggesting that BBB disruption may be related to the pathogenesis of AD. Research by Zenaro et al. [56] recently demonstrated the production of endovascular NETs in an AD model animal and suggested that this is one of the mechanisms of BBB disruption in AD. The potential mechanism proposed by the same author in a review is as follows [60]: A*β* activates cerebral endothelial cells, which express endothelial adhesion molecules, including intercellular cell adhesion molecule-1(ICAM-1) [56, 61]. Circulating neutrophils adhere to blood vessels by binding to ICAM-1 through the lymphocyte function associated antigen-1 (LFA-1) [56]. Adhered neutrophils secrete endovascular NETs, which may be associated with the binding of activated platelets to neutrophil LFA-1 via intercellular cell adhesion molecule-2 (ICAM-2) [56, 62] or triggered by proinflammatory cytokines such as tumor necrosis factor-*α* (TNF-*α*), interleukin-1*β* (IL-1*β*), and interleukin-8 (IL-8) released by activated endothelial cells [63–66]. Components of NETs such as NE and MMPs, myeloperoxidase (MPO) and histones could injure endothelial cells and promote BBB damage [45, 67–70]. Additionally, thrombosis caused by intravascular NETs could exacerbate cerebral amyloid angiopathy, which is another characteristic of AD caused by A*β* deposits. On the other hand, the discovery of NETs within the cerebral parenchyma of animal models and patients with AD [56] indicated that NETs represent a mechanism of intraparenchymal tissue damage in AD. Through LFA-1-ICAM-1 binding, neutrophils adhere to endothelial cells and infiltrate the brain parenchyma. By activating Nox in neutrophils, A*β* promotes the generation of ROS, potentially promoting intraparenchymal NETosis in AD [71]. Additionally, neutrophils in brain parenchyma may be activated by tumor necrosis TNF-*α*, IL-1*β*, and IL-8 secreted by glial cells and then releasing NETs [72–74], which may in turn activate glial cells and cause neuronal damage. Intraparenchymal NETs could cause neurotoxicity by disintegrating extracellular matrix, which could be caused by NET-associated proteases such as MMP-9, MPO, and NE, and activating the mitochondrial apoptosis pathway and amplifying the inflammatory process [75–78].

## 4. NETs in Autoimmune Diseases

Since extracellular histone complexes may serve as self-antigens that participate in the development of lupus erythematosus, it was recognized in the first report on NETs that the extracellular web-like structure may play a role in autoimmune diseases [14]. In fact, NETs are associated with many autoimmune diseases, including those that may affect the central and peripheral nervous systems (e.g., lupus erythematosus [26] and Behcet's disease [79]) and those that are specific to neural antigens such as multiple sclerosis (MS) [80].

MS is a chronic inflammatory, demyelinating disease of the CNS with a strong autoimmune component. Although the inflammatory cells associated with MS are mostly T lymphocytes cells and macrophages that accumulate within the CNS parenchyma and perivascular spaces, there is evidence that neutrophils also exert deleterious effects on the pathogenesis of MS [80]. The role of neutrophils in multiple sclerosis has been described in a recent review [81]: neutrophils can generate and present autoantigens, produce ROS, and release inflammatory mediators, enzymes, and NETs [82–85].

Although elevated levels of circulating NETs were identified in MS patients, early studies opposed a critical role of NETs in this disease, as increased circulating NET levels do not appear to be a common feature of relapsed remitting MS (RRMS) patients, and there was no correlation between the level of NETs in serum and disease activity [80, 86]. However, further investigation by these authors showed that MPO-DNA complexes were significantly higher in male patients, who generally suffer a worse prognosis, than in female patients [86], suggesting that high NET levels could underlie sex-specific differences in MS pathogenesis. In the same study, researchers also proposed the possibility that NETs adversely affect the BBB and induce damage to neighboring neurons in MS patients. Consistent with this hypothesis, there is evidence that the absence of NET-related proteins (MPO, NE) increases the integrity of the BBB and reduces severity of the disease [84, 87]. Furthermore, studies have shown that elevated plasma NE levels in patients with MS correlate with disease severity and clinical prognosis [88]. Researchers have demonstrated that NETs can activate inflammatory T helper 17 cells, which can secrete interleukin-17 (IL-17), a neutrophil-attracting cytokine. Depleting IL-17 to inhibit recruitment of neutrophils can significantly ameliorate the onset and severity of experimental autoimmune encephalomyelitis (EAE) [89], which is a rodent model of MS. However, there is still a lack of a strong direct association between NETs and MS lesions, and evidence that NETs cause the breakdown of the BBB and neuronal damage in MS patients still needs to be further explored. Paryzhak et al. suggested another possible effect of NETs, which is the degradation of circulating immune complexes in MS [90]. These authors emphasize the potency of NET-related proteases to cleave circulating IgG immune complexes, resulting in the exposure of hidden glycoepitopes, which may contribute to MS pathogenesis.

NETs may also be involved in neuropsychiatric lupus. Tay et al. [91] described a hypothesis for the pathogenesis of cognitive dysfunction in SLE. Elevated levels of MMP-9 in the serum of SLE patients may contribute to degeneration of the basal lamina and damage BBB integrity. Then, neutrophil recruitment, rolling, adhesion, migration, and intracerebral parenchymal NET release occur, which result from the activation of the cerebrovascular endothelium by anti-N-methyl-D-aspartate receptor subunit NR2A/B (anti-NR2A/B) autoantibodies. Finally, the formation of NETs leads to neurotoxicity by inducing neuronal death, resulting in cognitive disorders in SLE patients. Additionally, anti-NR2A/B autoantibodies cross the BBB into the CSF and may lead to disruption of neuronal cell activity via Ca2+ influx and subsequent activation of the apoptotic pathway, which may further cause CNS symptoms [92].

## 5. NETs in CNS Infection

It is well known that NETs can immobilize bacteria, kill bacteria with antimicrobial peptides, and restrict bacterial dissemination in the host. Recent research indicates the formation of NETs in the cerebrospinal fluid (CSF) of patients with pneumococcal meningitis, as well as patients with Lyme neuroborreliosis (LNB) and viral CNS infections such as enteroviral meningitis [93, 94]. However, the effect of NETs on CNS infections is far from clear.

In recent years, studies have reported that many bacteria, including *Streptococcus pneumoniae* [95, 96], *Staphylococcus aureus* [97] [98], and *Streptococcus suis* [99], can produce DNases to evade capture and killing by NETs, which facilitate their further dissemination. De Buhr et al. showed a balance in antimicrobial activity between NET evasion and NET stabilization [100]. Streptococcus suis- (S. suis-) secreted nuclease A (SsnA) and endonuclease A of S. suis (EndAsuis) were identified as NET evasion DNases that lead to the degradation of NETs in S. suis infection [99, 101]. However, despite a strong DNase activity was present in those CSF samples, NET fiber-trapping bacteria were still detectable in the CSF of the S. suis-infected piglets. These authors found that the antimicrobial peptides LL-37 and PR-39 play a role in preventing extracellular DNA from destroying nucleases, thereby restricting NET evasion mediated by nucleases in S. suis infection. It is hypothesized that the ultimate result of NET-mediated inhibition of S. suis rests in the balance between NET degradation caused by bacterial nuclease and NET stabilization via antimicrobial peptides.

A study by Mohanty et al. [93] showed the present of NETs in the CSF of patients with pneumococcal meningitis; however, the effect of NETs could run counter to their previously described antibacterial actions. Pneumococci are trapped in NETs without any decline in survival. They hide in the web-like structure of NETs and replicate and then pass through the damaged BBB and spread elsewhere. In a pneumococcal meningitis model rat, targeting extracellular web-like structures with DNase may unmask bacteria trapped in NETs and expose them to intact neutrophils, leading to bacterial death via neutrophil phagocytosis. The same effect was recently observed in piglets infected with S. suis. Meurer et al. [102] reported that host DNase 1 promoted the killing of S. suis by neutrophils by cleaving DNA fibers in NETs and facilitating neutrophil intracellular uptake and phagocytosis of bacteria. This finding was supported by the production of “vital” NETs, as previously described, in which neutrophils did not die after NET release. In summary, these authors suggested that NETs play a harmful role in pneumococcal meningitis and CNS infection with S. suis.

The evidence that certain bacteria are trapped and killed in the web-like framework of NETs, while others take advantage of NETs to avoid neutrophil phagocytosis, shows that NETs can be beneficial or detrimental in infectious diseases. At present, there have been few studies on the roles of NETs in CNS infections. Some studies have reported the harmful effects of NETs, but there have been no reports on the beneficial effects of NETs in CNS infection. Further investigation is warranted to reveal the exact roles of NETs in CNS infections.

## 6. NETs in CNS Tumors

Many studies have shown the presence of NETs in tumors, and many in vitro experiments have demonstrated that a variety of tumor cells can promote the formation of NETs [103, 104]. The role of NETs in tumors, however, remains complex. In malignant melanoma [105], NETs can come into contact with tumor cells and inhibit melanoma cell migration and viability. It is hypothesized that the antitumor effect of NETs is related to the ability of NETs to directly kill cancer cells or stimulate the immune system to fight against the tumor. In contrast, some studies hypothesized that NETs play a significant role in tumor proliferation, invasion, and metastasis [106–108]. Evidence shows that NETs can entrap circulating tumor cells and promote tumor cell adhesion and metastasis to distant organ sites [108].

The presence of NETs in tumors of CNS has rarely been reported. Early studies on the capacity of NET-related proteins such as elastase, proteinase-3, and cathepsin G to invade CNS tumors preliminarily showed traces of NETs in CNS tumors [109]. Recently, NETs were detected in the glioma tissue by staining for MPO and CitH3 [110]. Furthermore, by activating the NF-*κ*B signaling pathway through the binding of HMGB1 with receptor for advanced glycation end products (RAGE), NETs secreted by tumor-infiltrating neutrophils (TINs) participate in the proliferation and invasion of glioblastoma cells. Consistent with this observation, the levels of NETs in high-grade glioma tissues were significantly higher than those in low-grade glioma tissues. The author also described a positive feedback loop among neutrophils, NETs, and glioma cells. By activating the NF-*κ*B signaling pathway, NETs cannot only promote the progression of glioma but also induce the secretion of IL-8 by glioma cells. IL-8, which is widely known as a proinflammatory chemokine, can facilitate neutrophil infiltration into tumor sites. At the end of the cycle, the recruitment of neutrophils to glioma tissue can further result in NET production via the PI3K/AKT/ROS axis.

Salganik et al. [111] documented a reduction in the lymph node size and extended survival time at 12 weeks after injection of DNase I in spontaneous lymphatic leukemia mice. Other study have shown that injecting DNase I into laboratory animals can significantly inhibit pancreatic cancer cells invasion and metastasis [112].Therefore, future research should focus on whether the degradation of extracellular DNA fibers with DNase I can inhibit the proliferation and invasion of glioma cells.

## 7. NETs in Traumatic Brain Injury (TBI)

TBI is a transient or permanent neurological dysfunction caused by external forces. TBI is one of the main causes of disability and death in young people worldwide [113, 114]. In contrast to stroke, traumatic brain injury occurs more often in young adults than in older individuals [113, 114]. The high disability rate has brought huge social and economic burdens to individuals who are in the prime of their life. In addition to neuronal and widespread axonal damage caused by the initial trauma, subsequent elevated ICP, cerebral hypoperfusion, and inadequate tissue oxygenation can cause serious secondary lesions. Studies have shown that neutrophils enter the subarachnoid and subdural spaces within 4 hours of injury and penetrate into the parenchyma during the following days [115, 116]. By inducing edema formation and cerebral hypoperfusion, neutrophils play a significant role in experimental models of TBI [117, 118].

Vaibhav et al. [119] used scanning electron microscopy and observed an extracellular web-like framework within the controlled impact cortex in mice. CitH3 localized within infiltrated neutrophils in the cortex further corroborates the formation of NETs in the TBI brain. After TBI, CNS-infiltrated neutrophils exhibited elevated TLR4 expression, which correlated with poor TBI outcomes in patients [120]. The release of HMGB1 from necrotic neurons promoted cerebral edema via a TLR4–dependent mechanism, as observed in experimental TBI mice [121]. Vaibhav and colleagues found that NETs may exert their effects through TLR4, since they discovered that the activation of TLR4 promotes NETosis after experimental TBI, whereas mice lacking functional TLR4, exhibited less NET formation and displayed less edema development after TBI. Since a reduction in serum DNase-I activity is associated with elevated levels of circulating NETs in patients with severe nerve injury, researchers suggest that NETs may contribute to the development of cerebral edema and worsened neurological function in patients with TBI by means of correlation analysis of patient serum DNase activity and ICP or Glasgow Coma Scale (GCS) scores. The underlying mechanism may be that circulating NETs promote microthrombus formation, which leads to the obstruction of cerebral venous return, further resulting in cerebral edema. Finally, the researchers degraded both circulating and CNS-infiltrated NETs via intravenous administration of recombinant human DNase-I, which resulted in a decrease in cerebral edema.

## 8. Clinical Application Prospect of NETs

### 8.1. Reducing/Inhibiting the Formation of NETs to Treat/Prevent Diseases

Many of the studies on NETs, including those mentioned above, show the potential of degrading NETs to prevent and treat disease. As described previously, intravascular NETosis could play a critical role in atherothrombosis in IS patients with ICA occlusion [39], which suggests that combining traditional anticoagulant drugs with drugs targeting NETs may reduce the risk of thrombosis, thereby further preventing the occurrence of stroke. Previous studies have shown that the administration of DNase 1 to degrade NETs improves the efficacy of t-PA induced thrombolysis in vitro [37]. We expect that DNase 1 could be combined with fibrinolytic therapy, salvage the ischemic penumbra, and significantly improve the outcome of ischemic stroke patients. Minimally invasive surgery combined with alteplase has become a new strategy for the treatment of ICH in recent years [122]. A phase II clinical trial confirmed its safety, but the unsatisfactory hematoma clearance rate makes it difficult to further develop this treatment strategy [122, 123]. Tan et al. showed that degradation of the extracellular web-like framework by DNase 1 promotes t-PA induced hematoma fibrinolysis [49], which may provide a new strategy for minimally invasive surgery plus fibrinolysis therapy to treat ICH.

In addition to stroke, the administration of DNase in other NET-associated studies has achieved considerable results. For example, targeting NETs with DNase can alleviate secondary cerebral edema and improve cerebral perfusion in TBI, as mentioned previously. Furthermore, host DNase can unmask bacteria trapped in NETs and improve neutrophil phagocytosis of bacteria in pneumococcal meningitis and CNS infection with S. suis. Finally, cleavage of extracellular DNA fibers with DNase in glioma tissue can inhibit the proliferation and invasion of glioma cells, which also seemed promising. Intriguingly, a study reported that intermittent hypoxia-hyperoxia training (IHHT), which is a new nonpharmacological therapy, could inhibit NET formation and may enhance cognitive function in pre-AD patients and slow the progression of AD [124].

Based on the ability of NETs to fight against infection, we must consider whether a reduction in NETs after DNase administration could increase the risk of infection in critically ill patients. As described previously, PAD4 is a key protein that regulates the formation of NETs. There is evidence that PAD4-/- mice showed no increased susceptibility to severe bacterial infection, even though these mice could not produce NETs [125]. Furthermore, as a drug that has been approved by U.S. Food and Drug Administration, DNase 1 is already used for cystic fibrosis therapy and is a drug with an excellent record of clinical safety. These results indicate that DNase may have promising clinical applications in these NET-related diseases.

In addition to DNase 1, certain drugs or compounds have also been shown to inhibit or destroy NETs and may play therapeutic roles in CNS diseases. For example, aspirin, a nonsteroidal drug with antithrombotic and anti-inflammatory properties, can prevent NETosis by inhibiting the interactions between platelets and neutrophils [126, 127]. In patients with cerebral infarction who take aspirin orally, this potential effect may prevent the formation or further expansion of thrombosis. As mentioned previously, Zhou et al. demonstrated that NETs in carotid lesion sites of patients with ICA occlusion could induce the expression of PS and TF in endothelial cells and induce these cells to exhibit a procoagulant phenotype [39]. Sivelestat, a selective elastase inhibitor, can inhibit the cytotoxic effect of NETs by inhibiting elastase related to NETs, thus protecting endothelial cells, reducing procoagulant activity, and preventing thrombosis in patients with ICA occlusion [39]. Future treatment strategies can focus on the combination of drugs targeting NETs and classic antiplatelet drugs (although antiplatelet drugs such as aspirin and dipyridamole have also been proven to inhibit the formation of NETs) to further reduce the risk of thrombosis and reduce the dose of antiplatelet drugs.

Chlor-amidine is a nonspecific inhibitor of PAD that is capable of inhibiting PAD4 and reducing the formation of NETs [128]. As mentioned previously, PAD4 was significantly increased in the ischemic peripheral cortex, resulting in decreased neovascularization by releasing additional NETs [46]. Whether the formation of NETs can be inhibited by Chlor-amidine or other PAD inhibitors to increase neovascularization in the cortex around cerebral infarctions will be a future research topic.

HMGB1 plays an important role in ischemic cerebral infarction and can promote the generation of NETs and recruit immune cells to exacerbate neuroinflammation [43]. Studies have shown that the use of anti-HMGB1 antibodies can reduce NET formation [129, 130]. The application of an anti-HMGB1 antibody in ischemic cerebral infarction may reduce neuronal death after ischemia and improve subsequent neuroinflammation. Similarly, given that NETs can promote the proliferation and invasion of glioma though the binding of HMGB1 and RAGE [110], it is also possible that the administration of anti-HMGB1 antibodies to glioma patients can inhibit the deterioration and progression of tumors induced by NETs.

Surprisingly, metformin, a widely used hypoglycemic drug, has also been shown to reduce the concentration of NETs in vitro [131]. The potential mechanism of metformin may be related to the inhibition of Nox activation [131]. In Alzheimer's disease, A*β* peptides promote the production of ROS by activating neutrophil Nox, thus promoting NETosis in the brain parenchyma [71]. The application of metformin may inhibit this process to improve neurodegeneration in AD patients. In addition, it has been reported that metformin can downregulate the expression of ICAM-1 by activating AMP-activated kinase (AMPK) [132], which indicates that metformin also has the potential to reduce the formation of endovascular NETs in AD patients.

In addition, there are several other drugs or compounds that have been shown to inhibit NET release in vitro and could potentially reduce NET formation in the CNS (ticagrelor [133], colchicine [134], prostaglandins [135], ruxolitinib [136], etc.). However, no available treatment has been widely studied or approved for human administration to treat NET-associated CNS diseases, and more trials are needed for clinical translation. There are still many problems to be solved, such as whether these drugs can successfully cross the blood-brain barrier and quickly reach the lesion to exert an effect, whether the reduction in NETs will damage the immune system and cause serious infection, whether the side effects of the drugs themselves have been evaluated, and whether they need to be combined with other traditional treatment methods to achieve better treatment effects. In summary, there is a long way to go in treating CNS diseases through drugs that target NETs.

### 8.2. NETs as Circulating Markers

Clinical research by Lim et al. [137] demonstrated an elevated level of circulating dsDNA in patients with IS at the initial stage, suggesting that NETs could act as a novel circulating marker for the early diagnosis of IS. Furthermore, in a study of the plasma of IS patients, elevated levels of circulating CitH3, a specific biomarker of NETs, were independently associated with the severity and mortality of stroke [138]. All of the research mentioned above suggests that NETs can serve as novel circulating markers and may play an important role in the clinical diagnosis, early severity prediction, and prognosis estimation of IS.

The use of NETs as a new marker requires standardized studies of normal and abnormal levels, which involve measuring cell-free DNA (cfDNA), CitH3, NE, and other NET-related factors in the blood. The determination of cyclic MPO/cfDNA complexes and citH3 may be more suitable for NET analysis than the determination of cfDNA alone [139]. How to accurately and conveniently measure circulating NETs in the clinic needs further investigation.

## 9. Discussion

In recent years, the role of NETs in CNS diseases has captured a great deal of attention, and this topic has also been reviewed by other researchers [13]. In this review, we comprehensively reviewed and summarized the previously formulated concepts and supplemented the latest research data in recent two years. In addition, the clinical application prospect and the possibility of clinical transformation of NETs were also one of our focuses.

On the one hand, the research on the role of NETs in CNS diseases was unbalanced. The role of NETs in ischemic stroke has been widely studied; thus, we made a relatively comprehensive summary of this regard (Figure 2). However, the research on NETs in AD, MS, and CNS infection was inadequate, and more researches were required to illuminate the role of NETs in these diseases. In addition, recently published studies on NETs in cerebral hemorrhage, glioma, and TBI not only showed that NETs play a significant role in different CNS diseases but also displayed that the role of NETs in CNS diseases has been attached more attention. On the other hand, we summarized the clinical application prospect of NETs. Many drugs or compounds have been proved to inhibit the formation of NETs or degrade NETs through different mechanisms. We speculated that the application of these drugs or compounds can have beneficial effects on the related CNS diseases (whether in prevention or treatment), which was expected to be proved by subsequent studies. Additionally, NETs, served as circulating markers, may have the potential to predict the progression or prognosis of CNS diseases. Therefore, we hoped that this article can provide inspiration or useful information for the follow-up research of NETs in CNS diseases, so as to promote the progress of diagnosis and treatment of related CNS diseases.

## 10. Conclusion

There are growing evidences that NETs are present in many CNS diseases of different origins, in which they may play similar or different roles. In this paper, we described the roles of NETs in various brain diseases and explored their clinical application prospects. We expect that NETs can be detected clinically as circulating markers, and that targeting NETs can be used to treat certain CNS diseases. Since the role of NETs in CNS disease has been gradually explored, future research should focus on the similarities and differences in the role of NETs in CNS diseases and their clinical applications.

## Abbreviations

CNS: Central nervous system
BBB: Brain-blood barrier
MMPs: Matrix metalloproteinases
ROS: Reactive oxygen radicals
NETs: Neutrophil extracellular traps
PAD4: Peptidyl arginine deiminase 4
Nox: NADPH oxidase
NE: Neutrophil elastase
IS: Ischemic stroke
TLR4: Toll-like receptor 4
ICA: Internal carotid artery
PMPs: Platelet-derived microparticles
PS: Phosphatidylserine
TF: Tissue factor
MCAO: Middle cerebral artery occlusion
HMGB1: High-mobility group box-1
DAMP: Danger-associated molecular pattern
ICH: Intracerebral hemorrhage
AD: Alzheimer's disease
A*β*: Amyloid beta
ICAM-1: Intercellular cell adhesion molecule-1
ICAM-2: Intercellular cell adhesion molecule-2
MPO: Myeloperoxidase
MS: Multiple sclerosis
RRMS: Relapsed remitting MS
TNF-*α*: Tumor necrosis factor-*α*
IL-1*β*: Interleukin-1*β*
IL-8: Interleukin-8
IL-1: Interleukin-1
IL-17: Interleukin-17
anti-NR2A/B: Anti-N-methyl-D-aspartate receptor subunit NR2A/B
CSF: Cerebrospinal fluid
LNB: Lyme neuroborreliosis
S. suis: Streptococcus suis
SsnA: Streptococcus suis- (S. suis-) secreted nuclease A
EndAsuis: Endonuclease A of S. suis
CitH3: Citrullinated histone H3
RAGE: Receptor for advanced glycationend products
TINs: Tumor-infiltrating neutrophils
ICP: Traumatic brain injury (TBI); intracranial pressure
GCS: Glasgow Coma Scale
IHHT: Hypoxia-hyperoxia training
cfDNA: Cell-free DNA.
